# Focused abdominal ultrasound in preoperative liver surgery staging: a prospective study

**DOI:** 10.1186/1477-7819-11-138

**Published:** 2013-06-14

**Authors:** Marcela P Cohen, Paulo Herman, Rubens Chojniak, Miriam RB Poli, Paula NV Barbosa, Almir GV Bitencourt

**Affiliations:** 1 Department of Imaging, AC Camargo Cancer Center, Rua Professor Antônio Prudente, 211, São Paulo, Brazil; 2 Faculdade de Medicina, Universidade de São Paulo, Rua Eneas de Aguiar Carvalho, 255, São Paulo, Brazil

**Keywords:** Ultrasonography, Liver neoplasms, Preoperative care

## Abstract

**Background:**

Because of its safety, relative low cost and widespread availability, conventional ultrasound (US) is the modality of choice for initial evaluation of the liver. Following US, in patients eligible for surgery, further computed tomography and/or magnetic resonance imaging is usually recommended for surgical planning. There are no recent published series focusing on conventional abdominal US exclusively employed for the evaluation of liver nodules before surgery. The objective of this study is to evaluate the efficacy of focused conventional preoperative US in detecting liver lesions, and the impact of US findings on surgical management.

**Methods:**

Sixty-seven noncirrhotic patients underwent surgical resection, after being previously submitted to focused liver US evaluation. US results were compared with intraoperative US (IOUS) and histology (gold standard). The IOUS was performed by the same radiologist who performed the preoperative US. Patient-by-patient and lesion-by-lesion analyses were performed.

**Results:**

A total of 241 lesions were depicted in 67 patients. The mean number of lesions detected per patient by US and IOUS was 2.37 and 3.37, respectively (*P* = 0.001). In 52.2% of patients, US and IOUS depicted the same number of liver lesions. Surgery with curative intent was conducted in 61 (91.0%) patients. Histological evaluation was obtained in 196 lesions; 155 were considered malignant. The overall lesions detection rate by US was 65.6%. For lesions <15 mm and lesions ≥15 mm, US showed a sensitivity rate of 55.3% and 75.5%, respectively.

**Conclusions:**

The relatively high sensitivity rates achieved by US focused on liver evaluation, with the aim of lowering costs but not efficiency, places the method in focus again for use in the routine preoperative staging of candidates for liver resection. We suggest for preoperative evaluation that US could be associated with one section imaging method (computed tomography or magnetic resonance imaging) as routine.

## Background

Advances in imaging diagnosis, tumor staging, preoperative and postoperative care and surgical techniques have had a great impact on liver metastases resection in the last years. This impact is particularly evident in patients with colorectal cancer and neuroendocrine tumor metastases, and in selected cases with other neoplasms [1-3].

Higher survival rates are related to complete removal of hepatic metastases, and the success of the surgical treatment highly depends on preoperative staging [4]. Several imaging techniques are employed for preoperative staging of the liver and, in clinical practice, ultrasound (US), computed tomography (CT) and magnetic resonance imaging (MRI) are currently used for that purpose.

Because of its safety, relative low cost and widespread availability, conventional US is the modality of choice for the initial evaluation of liver resection candidates, and can be used to preclude from surgery patients with diffuse lesions. Following US, in patients eligible for surgery, further CT and/or MRI is usually recommended for surgical planning [5].

Although many series have been published concerning imaging diagnosis in focal liver lesions, mainly with CT and MRI, the optimal imaging staging strategy has not yet been defined, which means that there is no agreement about the best imaging strategy for liver surgical planning.

Intraoperative ultrasound (IOUS) is well established as the best imaging modality for liver lesion depiction and, in some series, is considered the gold standard for liver evaluation [6-8].

The real place for US in liver staging has not been established. Besides the relevant advantages of US, there are drawbacks related to the operator, patient and equipment. As a result, US examination has a wide range of sensitivity rates according to the published series. Low sensitivity rates are usually related to studies performed previous to state-of-the-art equipment and, there are no recent published series focusing on conventional abdominal US exclusively employed for the evaluation of liver nodules before surgery.

This study seeks to determine whether US examination made exclusively for liver evaluation, using modern equipment and operated by experienced radiologists, may contribute to liver staging before hepatic resection. In order to answer this question, the purpose of the study was to evaluate the efficacy of conventional preoperative abdominal US in detecting liver lesions, and the impact of US findings on surgical management.

## Methods

### Patients

From March 2002 to June 2007, 67 patients were studied. After being indicated for curative intent surgery by a multidisciplinary team, patients were sent to the Imaging Department for US liver evaluation.

Patients were included in this study if preoperative US had been performed within 14 days before surgery and cirrhotic patients were excluded. All patients were informed about the study and signed an informed consent.

There were 34 men and 33 women, with a mean age of 57 years (range: 20 to 73 years). Diagnosis included colorectal cancer metastasis (*n* = 51), neuroendocrine tumor metastasis (*n* = 6), peripheral cholangiocarcinoma (*n* = 3), gastrointestinal stromal tumor metastasis (*n* = 2), one melanoma metastasis, one basal cell carcinoma metastasis, one gastric cancer metastasis, one breast cancer metastasis and one bladder cancer metastasis.

### Ultrasound

US examination was performed using ATL HDI-5000 equipment (Philips Medical Systems, Bothel WA, USA) with a convex 2 to 5 mHz probe and, when necessary, a linear 5 to 12 mHz probe and Doppler duplex. US was performed at the dorsal and left lateral decubitus, through an intercostal and subcostal approach with a breath-hold maneuver. Two experienced radiologists, not blind to previous clinical and radiologic data, performed the US and gave the results in consensus.

US findings were recorded by number, size and location of the lesions. Typical hemangioma (<3 cm homogeneous hyperechoic and well delimited) and cystic lesions (anechoic lumen, increased through transmission and well-defined wall) were excluded from the analysis. Any lesion not meeting those criteria was included in the analysis.

The number, size and location of liver lesions were also recorded according to the IOUS and, for removed liver segments, histological findings.

The lesion’s location was established according to the Brisbane classification: left lateral section (Couinaud’s segments II and III), left medial section (Couinaud’s segment IV), right anterior section (Couinaud’s segments V and VIII) and right posterior section (Couinaud’s segments VI and VII). Any lesion >40 mm or occupying more than one of these segments was considered separately.

### Surgical plan

The surgical plan was first established at the multidisciplinary meeting, and, if necessary, modified after the conventional liver US. The definitive surgical strategy was established after palpation and IOUS evaluation. The IOUS was performed by the same radiologist who performed the preoperative US, using Tosbee equipment (Toshiba, Tokyo, Japan) with a T-shaped 7 mHz intraoperative probe bound in sterile plastic.

### Data analysis

Results were evaluated with reference to lesion-by-lesion and patient-by-patient databases and US results were compared with IOUS and histological evaluation results. Histology was considered the gold standard.

Based on the patient-by-patient analysis, surgical planning changes were analyzed, and the total number and mean number of lesions detected per patient by US and by IOUS were compared (by Wilcoxon test and intra-class correlation coefficient).

Based on lesion-by-lesion analysis, the sensitivity, specificity and positive predictive values were calculated for US and IOUS using histological findings as the gold standard. The agreement rate between US and IOUS was calculated by the Kappa test.

Despite not being the main objective of the study, the nonstandardized CT and MRI examinations previously carried out were nevertheless analyzed and the results compared with those of US, since the preoperative surgical decision was based on them. Good quality examinations made within 40 days before surgery were considered for analysis: CT scans with multidetector or spiral equipment with oral and intravenous iodine contrast acquired at pre-contrast, arterial phase and portal venous phase with a maximum of 5 mm thickness section were accepted. MRI examinations acquired using high magnetic field equipment with T1-weighted sections, before and after paramagnetic contrast administration and dynamic acquisition, with in-phase and out-of-phase sequences, and T2-weighted images obtained with and without fat-saturation sequences were accepted.

## Results

### Patient-by-patient analysis

**Figure 1:**
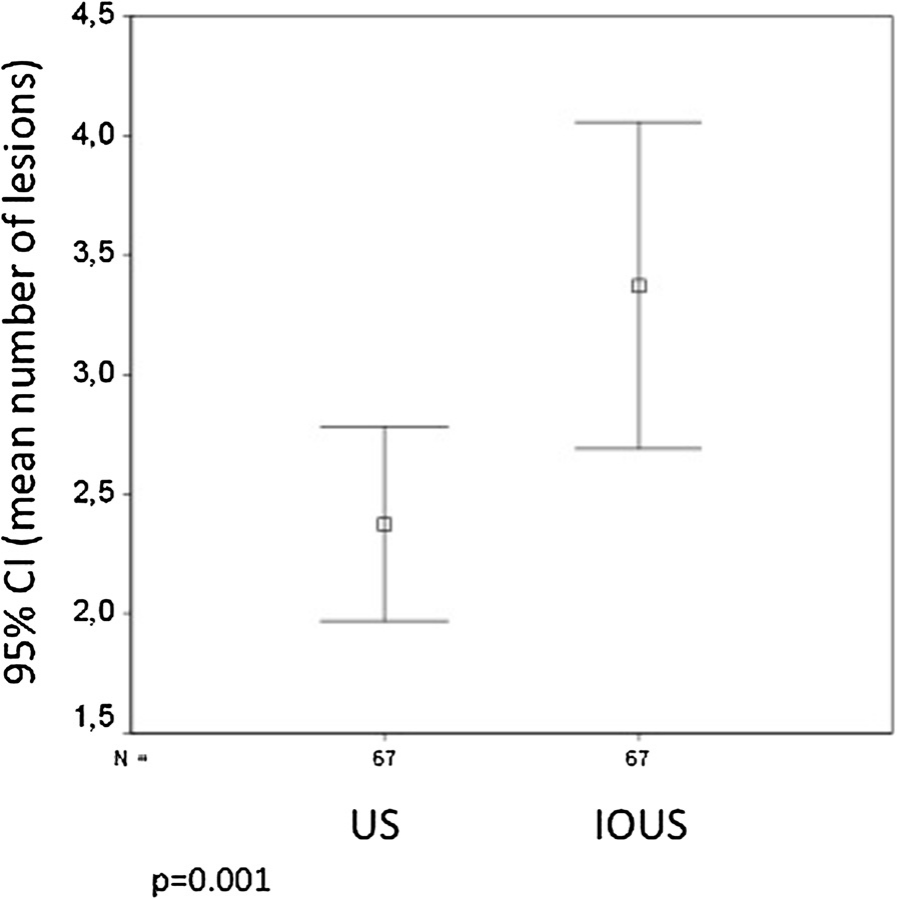
**Number of lesions detected per patient by ultrasound and by intraoperative ultrasound.** Mean and 95% confidence interval (CI) for number of lesions detected per patient by ultrasound (US) and by intraoperative ultrasound (IOUS).

**Figure 2:**
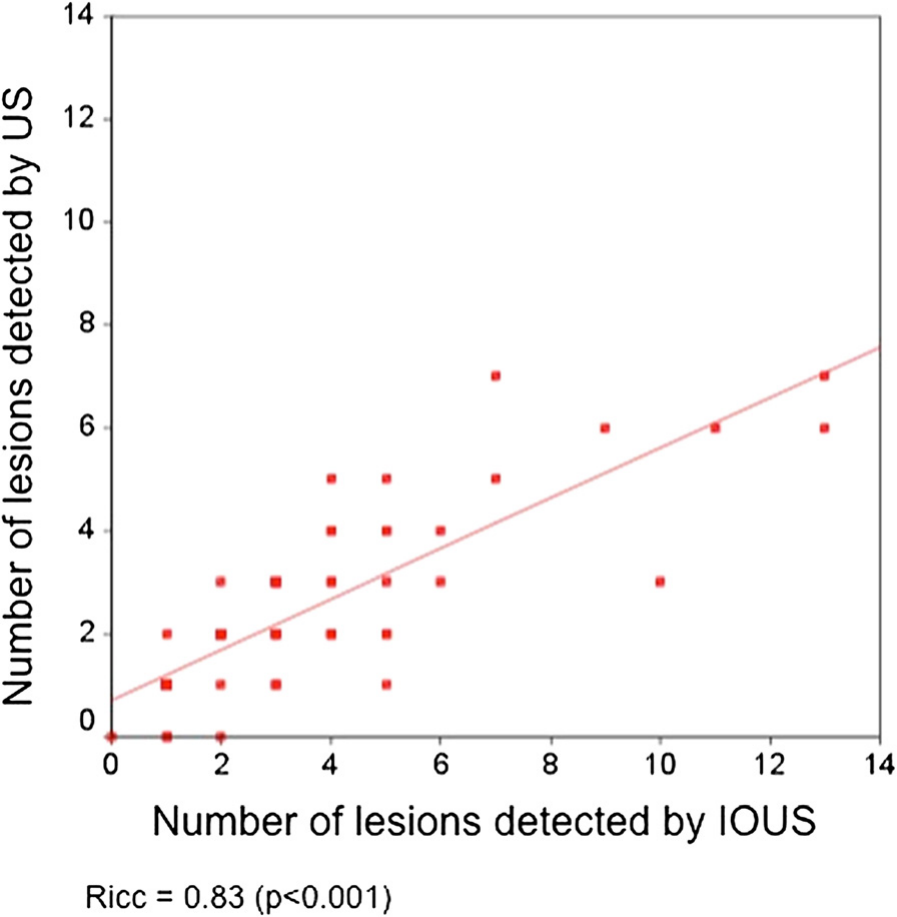
**Intra-class correlation coefficient for number of lesions detected per patient by ultrasound and intraoperative ultrasound.** Intra-class correlation coefficient (Ricc) for number of lesions detected per patient by ultrasound (US) and by intraoperative ultrasound (IOUS).

Sixty-seven patients were included in the study. The mean number of lesions detected per patient by US and IOUS was 2.37 and 3.37, respectively (*P* = 0.001; Figure 1). In 52.2% of patients, US and IOUS depicted the same number of liver lesions. In 4.5% of patients, US detected more lesions than IOUS; and in 43.3% of patients, the IOUS depicted from one to seven lesions in addition to the preoperative US results. The intra-class correlation index for US and IOUS was 0.83 (*P* <0.001; Figure 2).

There was no statistically significant difference in the number of lesions detected by US and by IOUS concerning the presence of steatosis (in US or histology), prior chemotherapy or body mass index.

Surgery with curative intent was conducted in 61 (91%) patients, including two IOUS-guided radiofrequency ablations. In six patients, intraoperative findings precluded liver resection; one of these patients had melanoma and intraoperative inspection depicted diffuse peritoneal lesions not detected by previous US, CT or MRI. In three patients there were hilar lymph node metastases; in another patient, IOUS accurately identified hepatic vein invasion, precluding resection. Vascular invasion suspicion had already been depicted by the preoperative US. In the last patient, US identified diffuse small liver lesions while CT detected two lesions and positron emission tomography–CT identified only one uptake lesion. Because of these conflicting results, the patient underwent surgical exploration that confirmed preoperative US findings, precluding resection.

The surgical plan, based on previous imaging examinations (CT and/or MRI and US), was modified in 17 (25.5%) patients after the IOUS. Most of these changes consisted of a new strategy to the previous planned hepatectomy (due to new or more precisely located lesions given by IOUS).

### Lesion-by-lesion analysis

Two-hundred and forty-one lesions were depicted in 67 patients (mean 3.4; range 1 to 13 lesions per patient) with a mean diameter of 24.1 mm (median 17 mm; range 2 to 120 mm).

Histological evaluation was obtained in 196 lesions; 155 were considered malignant (151 metastases and four liver primary tumors) and 41 (20.9%) benign (Table 1).

**Table 1:** Number of lesions according to histological diagnosis

**Histological diagnosis**	**Number of lesions (%)**
Metastases	151 (77.0)
Cholangiocarcinoma	3 (1.5)
Hepatocellular carcinoma	1 (0.5)
Normal liver	14 (7.1)
Steatosis	10 (5.1)
Fibrosis / necrosis (post chemotherapy)	13 (5.6)
Hyperplastic nodule	3 (1.5)
Hemangioma	1 (0.5)
Focal nodular hyperplasia	1 (0.5)
Biliary hematoma	1 (0.5)
Total	196 (100)

Considering the 155 malignant lesions, 24.5% and 4.5% were not depicted by US and by IOUS, respectively.

The overall lesion detection rate by US was 65.6% (158 of 241; 95% confidence interval: 62.2%, 74.0%), while by nonstandardized CT the rate was 64.8% (46 of 72; 95% confidence interval: 56.0%, 78.0%) and by nonstandardized MRI it was 56.3% (40 of 71; 95% confidence interval: 59.9%, 61.3%).

Considering 196 lesions submitted to histological evaluation, the sensitivity of US, IOUS, CT and MRI was calculated taking into account the size of the lesions and using histology as the gold standard (Table 2).

**Table 2:** Sensitivity of ultrasound, intraoperative ultrasound, nonstandardized computed tomography and magnetic resonance

	**Sensitivity, % (number of lesions)**		
**Imaging method**	**All lesions**	**Lesions <15 mm**	***P *value**
Ultrasound	75.5 (196)	55.3 (96)	0.0050
Intraoperative ultrasound	95.5 (196)	92.3 (96)	0.5136
Computed tomography	75.5 (67)	33.3 (23)	0.0131
Magnetic resonance imaging	63.6 (55)	31.5 (26)	0.0391

Sensitivity using histological evaluation as the gold standard considering separately all lesions and lesions <15 mm.

The sensitivity rates of imaging methods were lower for lesions <15 mm, with a statistically significant difference (US: 55.3% × 75.5%, *P* = 0.005; CT: 33.3% × 75.5%, *P* = 0.013; MRI: 31.5% × 63.6%, *P* = 0.039).

Indeed, for lesions <15 mm, US showed a higher sensitivity rate when compared with the nonstandardized CT and MRI examinations, although this was not statistically significant (US, 55.3%; CT, 33.3%; MRI, 31.5%; *P* = 0.28 (US × CT) and *P* = 0.12 (US × MRI)).

## Discussion

Preoperative imaging techniques play an important role in patient selection and surgical planning for liver resection. According to several authors, US is recommended as the first imaging method for liver lesion detection [9]. For further surgical planning, CT and MRI are the most frequently employed imaging methods; however, there is no agreement for the best preoperative approach. A multi-modality strategy is therefore recommended, since no single modality can accurately detect all liver tumors.

Although limitations related to equipment and operator dependence are drawbacks for US examination, this method yields important advantages due to lack of risks, low cost and availability. Over the last 10 to 15 years, however, there have been few published studies focusing on the efficacy of US on liver lesion detection or on its contribution to liver resection strategy [5,9]. Furthermore, published series describing low US sensitivity rates usually rely on the use of nonstate-of-the-art equipment [10].

For more than one-half of the patients (52.2%) included in this study, the same number of lesions was detected by US and IOUS. Konopke and colleagues presented similar results employing contrast-enhanced US, with 50% of patients on their series with identical findings for both examinations [11]. These authors achieved lower sensitivity rates for conventional US, comparable with our findings.

Higher sensitivity rates related to conventional US were achieved by Dietrich and colleagues (84.6%) and Albrecht and colleagues (71%). These authors used other imaging methods, such as CT and MRI, rather than histology or IOUS as the gold standard, which might have had an impact on sensitivity rates [12,13].

The high sensitivity rate achieved by IOUS (95.5%) and its higher lesion detection rate per patient when compared with US are expected results according to other studies [6-8]. In the present study, curative intent surgery was achieved in 91% of patients, a result in accordance with other series, with 87 to 94.1% curative intent surgery [14,15].

Although not the main objective of this study, nonstandardized CT and MRI were compared with US and IOUS. We did not find any statistically significant difference between the sensitivity rates of these examinations and preoperative US. While US was performed by two experienced radiologists using state-of-the-art equipment, not blinded to previous imaging examinations, CT and MRI scans were performed with nonstandardized protocols or equipment. Indeed, although not ideal, the evaluation of nonstandardized CT and MRI represents the daily practice of a liver surgeon, who receives patients that have already been submitted to radiological evaluation and, based on these results, establishes the surgical plan. For small hepatic lesions (<15 mm), US presented higher sensitivity rates (55.3%) than CT (33.3%) and MRI (31.5%), although no significant difference was observed.

Considering that multiple imaging studies are recommended for liver staging, Ward and colleagues suggested that CT and MRI should both be employed routinely on preoperative evaluation [16]. We suggest for preoperative evaluation that US could be associated with one section imaging method (CT or MRI) as routine, instead of the association of CT and MRI.

## Conclusions

The relatively high sensitivity rates achieved by US when performed by experienced radiologists and focusing only on liver evaluation, with no significant difference when compared with nonstandardized CT and MRI in the present series, places the method in focus again for new studies, with the aim of lowing costs but not the efficiency, in the routine preoperative staging of liver resection candidates. This study confirms that the best treatment option for a patient with a liver tumor is to be studied by a specialized team and radiologist with focused interest in liver tumors. The imaging method is probably of secondary interest.

## Abbreviations

CT: Computed tomography; IOUS: Intraoperative ultrasound; MRI: Magnetic resonance imaging; US: Ultrasound.

## Competing interests

The authors declare that they have no competing interests.

## Authors’ contributions

MPC, PH and RC conceived the study and participated in its design and coordination. MPC, MRBP and PNVB participated in acquisition and interpretation of data and helped draft the manuscript. MPC and AGVB performed the literature review and data analysis, and drafted the manuscript. PH and RC revised the manuscript. All authors read and approved the final manuscript.
